# Inactivation of Opportunistic Pathogens *Acinetobacter baumannii* and *Stenotrophomonas maltophilia* by Antimicrobial Photodynamic Therapy

**DOI:** 10.3390/microorganisms10030506

**Published:** 2022-02-25

**Authors:** Irina Buchovec, Laurita Klimkaitė, Edita Sužiedėlienė, Saulius Bagdonas

**Affiliations:** 1 Institute of Photonics and Nanotechnology, Faculty of Physics, Vilnius University, LT-10257 Vilnius, Lithuania; 2 Institute of Biosciences, Life Sciences Center, Vilnius University, LT-10257 Vilnius, Lithuania; laurita.klimkaite@kc.vu.lt (L.K.); edita.suziedeliene@cr.vu.lt (E.S.); 3 Laser Research Center, Faculty of Physics, Vilnius University, LT-10257 Vilnius, Lithuania; saulius.bagdonas@ff.vu.lt

**Keywords:** *Acinetobacter baumannii*, *Stenotrophomonas maltophilia*, photodynamic therapy, biofilms, natural photosensitizers, riboflavin, chlorophyllin, photostability

## Abstract

*Acinetobacter baumannii* and *Stenotrophomonas maltophilia* are opportunistic pathogens causing hospital infections with limited treatment options due to bacterial multidrug resistance. Here, we report that antimicrobial photodynamic therapy (aPDT) based on the natural photosensitizers riboflavin and chlorophyllin inactivates *A. baumannii* and *S. maltophilia*. The riboflavin and chlorophyllin photostability experiments assessed the photomodifications of photosensitizers under the conditions subsequently used to inactivate *A. baumannii* and *S. maltophilia*. *A. baumannii* planktonic cells were more sensitive to riboflavin-aPDT, while biofilm bacteria were more efficiently inactivated by chlorophyllin-aPDT. *S. maltophilia* planktonic and biofilm cells were more susceptible to chlorophyllin-aPDT compared to riboflavin-aPDT. The results suggest that riboflavin- and chlorophyllin-aPDT can be considered as a potential antimicrobial treatment for *A. baumannii* and *S. maltophilia* inactivation.

## 1. Introduction

Infections caused by antibiotic-resistant bacteria are a global problem urgently requiring new antibacterial agents or alternative antibacterial strategies [1]. During the last decade, the important clinical concern worldwide became nosocomial infections caused by Gram-negative opportunistic pathogens resistant to most clinically used antibiotics [2,3]. Opportunistic infections are hazardous to patients with compromised immunity, and are characterized by high morbidity and mortality [4]. The bacterium *Acinetobacter baumannii* is among the top infection agents for which new antimicrobial therapies are needed [5]. Genome plasticity and horizontal gene transfer enable successful *A. baumannii* adaptation to the clinical environment, the development of antibiotic resistance and virulence [6]. The prevalence of carbapenem-resistant *A. baumannii* in Europe reached 80% [7,8], creating the challenge of addressing these pathogen infections. Another newly emerging Gram-negative opportunistic pathogen causing serious concern is *Stenotrophomonas maltophilia* [9,10]. Although this bacterium is commonly found in nature, it also becomes dangerous to patients with deficient immunity [11,12]. *S. maltophilia* exhibits numerous virulence factors [13], as well as resistance to multiple antimicrobial agents [14]. Susceptibility against trimethoprim/sulfamethoxazole, the first-choice treatment for *S. maltophilia* infections, generally remains high (varying from 79 to 96%). However, mortality rates for patients with resistant *S. maltophilia* infections can range from 14 to 69% [15].

Among the important virulence features responsible for pathogen survival and spread in a hospital environment is their ability to form biofilms on abiotic (medical equipment) and biotic (tissues and cells of the host) surfaces [16]. Bacteria in biofilm structures are surrounded by an extracellular matrix consisting of polysaccharides, DNA and proteins. Therefore, biofilms are significantly more resistant to antibiotic treatment, disinfectants, physical stress and host immunity [17]. Clinical *A. baumannii* and *S. maltophilia* isolates are characterized by their strong ability to form biofilms [13,18]. Mechanical, chemical and physical methods are used for biofilm control, but they all have limitations. Therefore, there is a need for more efficient approaches to biofilm inactivation [19].

Antimicrobial photodynamic therapy (aPDT) is a modern biophotonic technology which can be used as a viable alternative for inactivating antibiotic-resistant pathogens [20]. aPDT is based on the interaction of a photosensitizer (PS), molecular oxygen and low doses of light applied at suitable spectral region to match the PS absorption peak [21,22]. Usually, after light excitation, the triplet-state of PS interacts with molecular oxygen, electron donors or electron acceptors, and can produce reactive oxygen species (ROS), thereby triggering photo-oxidative reactions which initiate various types of cellular damage and can destroy bacterial cells [23]. The efficacy of aPDT depends on many factors, but most significantly on the photophysical properties of the used PS. PSs can be divided into several main groups based on their structure and origin—synthetic dyes, nanostructures, and natural PSs [24]. Natural PSs, such as riboflavin (RF) and chlorophyllin (Chl), are safe and environmentally sustainable [22]. However, data on natural PS-based aPDT perspectives in the control of emerging multidrug resistant hospital infection agents and their biofilms remain scarce. For *A. baumannii* aPDT inactivation, only a few natural PSs [25,26,27,28] have been analyzed, although none of these studies addressed biofilm inactivation. Furthermore, no research has been performed on *S. maltophilia* aPDT inactivation, neither with natural nor with other PSs. In this study we aim to investigate whether natural PS riboflavin- and chlorophyllin-based aPDT can efficiently inactivate antibiotic-resistant *A. baumannii* and *S. maltophilia* bacteria and their biofilms.

## 2. Materials and Methods

### 2.1. Solutions

For the aPDT experiments, a stock solution of riboflavin (RF) (MW = 376.36 g/mol, Sigma-Aldrich, St. Louis, MO, USA) (0.11 mM, pH 6.8) was prepared by stirring RF in distilled water at 50 °C for 4 h [29]. The solution was sterilized by a 0.22 µm syringe filter and stored at 4 °C in the dark before use. A stock solution of non-copperized chlorophyllin sodium salt (Chl) (MW = 684.9 g/mol, Carl Roth, Karlsruhe, Germany) (1.5 mM, pH 7.2) was prepared by pipetting Chl at room temperature for about 60 s without any heating or shaking [30]. All working solutions were freshly prepared by diluting them with 0.01 M PBS buffer on the day of use.

### 2.2. Bacterial Strains and Growth Conditions

*A. baumannii* clinical isolate II-a [31] and *S. maltophilia* clinical isolate SM3 [32] were chosen for the aPDT approach study. In all experiments, *A. baumannii* was grown in Luria-Bertani (LB) and *S. maltophilia* was grown in Tryptone soya broth (TSB) medium; both isolates were grown at 37 °C.

### 2.3. Light Source for aPDT

**Figure 1:**
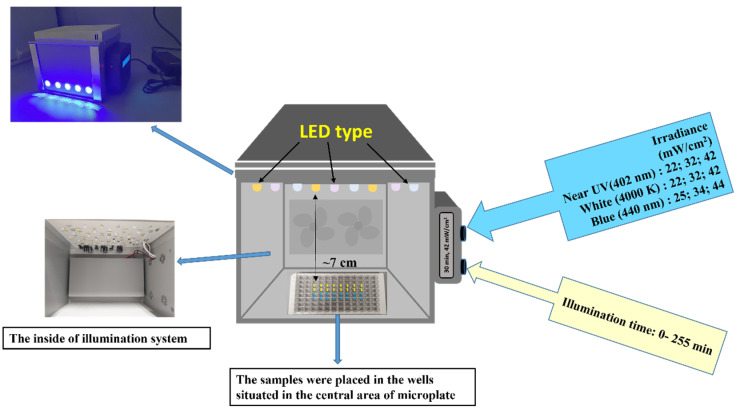
The illumination system used in the aPDT experiments.

The LED-based light source for the photoinactivation of bacteria was constructed at the Institute of Photonics and Nanotechnology of Vilnius University (Figure 1). It consisted of an illumination chamber and a supply unit. The illumination chamber size was optimized for even illumination of microplates (96 wells) or Petri dishes by the light of a selectable predefined spectrum.

**Figure 2:**
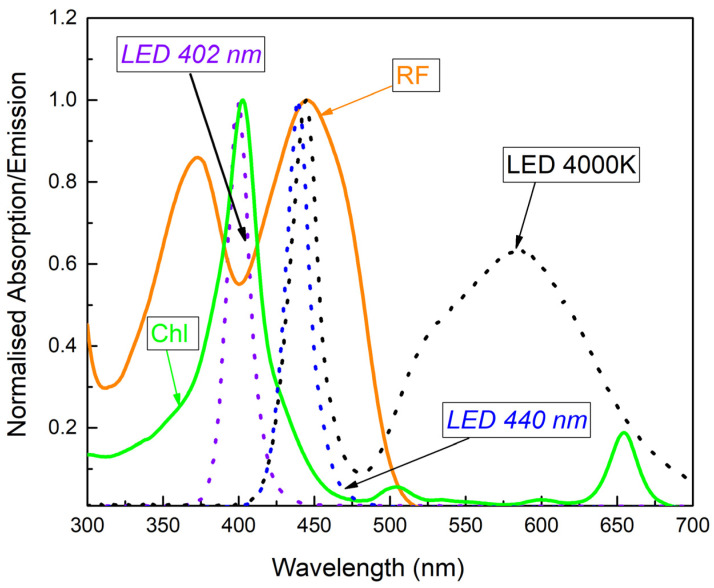
Normalized absorption spectra of RF and Chl in 0.01 M PBS, as well as emission spectra of the selected 402 nm and 440 nm LED. LED 4000 K was not used in the study.

The illumination routines and timing were controlled by an integrated microcontroller unit (MCU), which comprises three types of LEDs emitting at the near-UV (402 nm), blue (440 nm), and white (4000 K) spectral regions. Two types of LEDs (402 nm and 440 nm) with emission peaks near the maximum absorption of RF and Chl were used (Figure 2). 

The light irradiance at the surface of the samples (7 cm from the light source) reached 25, 42 and −4 mW/cm^2^. A cooling system was integrated into the light source to dissipate heat and minimize the heating of the sample. The illumination dose (J/cm^2^) was calculated as irradiance (mW/cm^2^) multiplied by illumination time (s). The illumination doses used in experiments are shown in Table 1.

### 2.4. Spectrophotometric Assessment

The absorption spectra of the PSs solutions were recorded by means of a LAMBDA 950 UV-VIS-NIR spectrophotometer in the spectral region of 300–600 nm and 300–700 nm (for RF and Chl, respectively). Polymethyl methacrylate cuvettes of 1 cm were used for the measurements. All measurements were performed at 20 ± 2 °C. The solutions of 0.011 mM RF (pH 7.4) and 0.015 mM Chl (pH 7.4) in 0.01 M PBS were illuminated with an LED-based light source for analysis of the photostability of the PSs. The changes in the absorption spectra of RF and Chl were investigated after LED illumination at 440 nm or 402 nm. For this purpose, 200 µL of the PSs were transferred to microplates (16 wells) and exposed to 42 and 44 mW/cm^2^ irradiance. After each illumination dose was applied separately, the samples (3 mL) were collected into cuvettes and used for spectrophotometric measurements.

### 2.5. Effect of aPDT on the A. baumannii and S. maltophilia Planktonic Cells

The overnight cultures of *A. baumannii* and *S. maltophilia* were diluted 1000 times with a fresh medium and grown until reaching OD600 = 0.65 for *A. baumannii* and OD600 = 0.45 for *S. maltophilia* (1–5 × 10^8^ colony forming units (CFU)/mL). Then, the bacteria were harvested by centrifugation (10 min, 6 °C, 7000× *g*), suspended in the 0.01 M PBS and immediately used for the riboflavin-based aPDT (RF-aPDT) and chlorophyllin-based aPDT (Chl-aPDT) experiments using LED illumination at 440 nm and 402 nm, respectively. The bacterial cultures (~1 × 10^7^ CFU/mL) were suspended with 0.011 mM RF or 0.015 mM Chl in the dark at room temperature. For the photoinactivation, 200 μL of the samples (control and with PSs) were placed into sterile, flat-bottom wells and exposed to LED light for different periods (Table 1). Light and dark controls of *A. baumannii* and *S. maltophilia* were also included, and some were incubated in the dark (0 ÷ 30 min) while the other samples were irradiated. The antibacterial effects of aPDT were evaluated by the microdilution method [33], where 10 μL of appropriate dilutions of bacterial test culture after treatment was applied to a separate LB plate and incubated at 37 °C for 24 h. After incubation, the colonies were counted and an average value was calculated for every point (from 3 to 6 experiments) and expressed as log of CFU/mL.

### 2.6. Inactivation of A. baumannii and S. maltophilia Biofilms with aPDT

For biofilm formation, overnight *A. baumannii* II-a and *S. maltophilia* SM3 cultures were diluted 1000 times and 100 μL aliquots (1–5 × 10^7^ CFU/mL) were transferred to sterile 96-well microtiter polystyrene plates and incubated at 37 °C for 24 h. After the incubation, the medium was discarded and the wells were gently washed three times with sterile PBS (pH 7.4). For aPDT, biofilm wells were filled with 200 µL of 0.11 mM RF or 0.15 mM Chl solutions. The control wells contained 200 µL of PBS. To maximize PS adsorption to the biofilms, the plates were incubated in the dark at room temperature (aprox. 25 °C) for 60 min. When illumination was not applied, the biofilms were detached immediately after incubation. For aPDT of biofilms with RF or Chl as well as in the case of the control, the wells were illuminated for 60 min with 440 nm LEDs (44 mW/cm^2^) or 402 nm LEDs (42 mW/cm^2^) appropriate for RF and Chl, respectively. After illumination, bacterial biofilms were mechanically detached from the wells and vigorously vortexed. aPDT efficacy against *A. baumannii* and *S. maltophilia* biofilms was evaluated by determining the CFU on LB plates (microdrop method), as described previously [33].

### 2.7. Statistical Analysis

The aPDT inactivation experiments were repeated at least 3–6 times. A standard deviation was estimated for every experimental point, as marked in the figure as an error bar. Data were processed with Origin Pro 9.1 software (OriginLab Corporation, Northampton, MA, USA) and were statistically analyzed using One-way Analysis of Variance (ANOVA). The post hoc Bonferroni test was used for the comparison between the experimental groups and the control group. The level of significance was set at *p* < 0.05.

## 3. Results

### 3.1. Photostability of Riboflavin and Chlorophyllin

Almost all PSs are degraded during the light illumination through oxygen mediated processes. Therefore, it is important to assess PSs photostability. Fast photobleaching can be a disadvantage in aPDT, as the PS cannot form enough ROS to kill bacteria. To assess photostability under different illumination doses, we analyzed the changes in the absorption characteristics of riboflavin (RF) and chlorophyllin (Chl), after illumination with 402 nm or 440 nm LED light.

**Figure 3:**
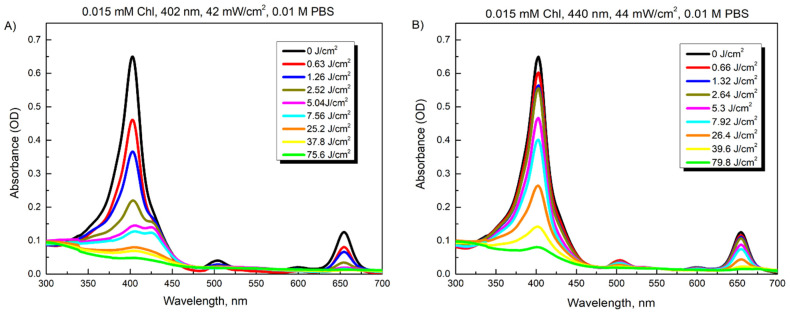
Changes in absorption spectra of 0.015 mM Chl after increasing illumination with 402 nm (**A**) and 440 nm (**B**) light.

Figure 3A shows the spectra recorded after illumination of 0.015 mM Chl solution in PBS at 42 mW/cm^2^ for different time periods (0–30 min, which corresponds to 0–75.6 J/cm^2^ illumination doses). The absorption peak of Chl at 403 nm rapidly decreased to about 20% after illumination for 3 min (7.56 J/cm^2^) and reached 12% of its magnitude after 10 min (25.2 J/cm^2^). The illumination at 440 nm diminished the peak magnitude to the same extent only after 20–30 min (79.8 J/cm^2^), which was consistent with the reduced absorbance intensity of the Chl in this spectral region (Figure 3B).

**Figure 4:**
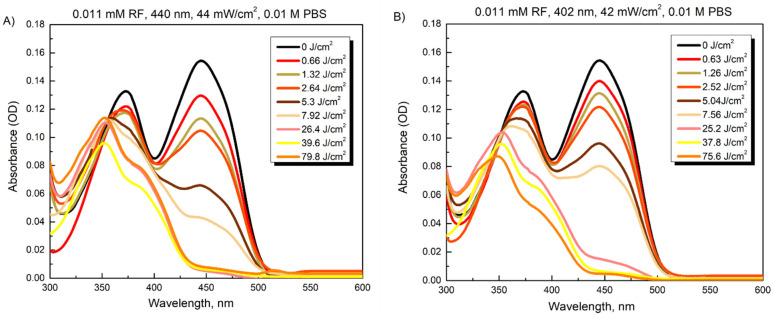
Changes in absorption spectra of 0.011 mM RF after exposures with 440 nm (**A**) and 402 nm (**B**) light.

Figure 4 illustrates the changes in spectra of the 0.011 mM RF solution after illumination with 440 nm (44 mW/cm^2^) or 402 nm light (42 mW/cm^2^) for various periods (0–30 min, which correspond to illumination doses of 0–79.8 J/cm^2^). The absorbance of RF at 373 and 444 nm decreased unevenly after LED illumination at 440 nm for 2 min (5.3 J/cm^2^) (Figure 4A). Notably, the absorption band at 444 nm vanished after 10 min (26.4 J/cm^2^). The same decrease in intensity of the main absorption band was observed only after 15 min of LED illumination at 402 nm. Meanwhile, the absorption changes induced at 373 nm under these illumination doses showed opposite tendencies for two used LED types. In contrast to Chl photodegradation, these spectroscopic data revealed the photomodification of the RF in buffered solutions at pH 7.2. Thus, after illumination doses of 26.4 J/cm^2^ and 37.8 J/cm^2^ at 440 nm or 402 nm, respectively, the main absorption peak at 444 nm disappeared, while a second peak at 373 nm transformed into one at 353 nm. Such spectral features imply that the RF underwent phototransformation to a lumichrome photoproduct [34,35,36].

These experiments also allowed us to test the phototransforming efficacy of the light from two different LEDs being applied on both PSs at chosen irradiances in order to set the appropriate conditions for the comparison of the antibacterial activity of Chl and RF. Based on the obtained photostability data, the highest illumination doses were determined in the case of each light source.

### 3.2. Inactivation of Planktonic A. baumannii and S. maltophilia Cells with aPDT

**Figure 5:**
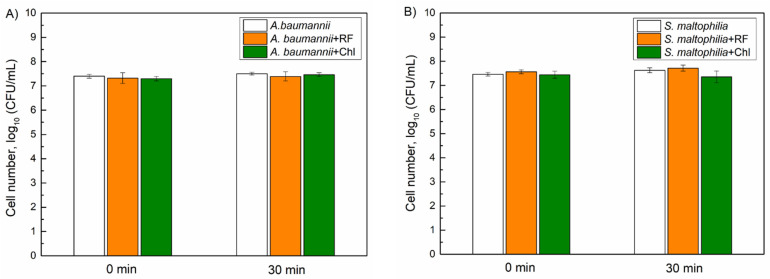
Dark toxicity of 0.015 mM chlorophyllin (Chl) and 0.011 mM riboflavin (RF) on the viability of *A. baumannii* (**A**) and *S. maltophilia* (**B**) planktonic cells. CFU values present the average of 3–6 experiments, error bars indicate standard deviation.

First, we evaluated the dark toxicity of Chl and RF on the viability of *A. baumannii* and *S. maltophilia*. Planktonic bacterial cells were dark-incubated with 0.015 mM of Chl and 0.011 mM of RF for different time periods, and then viability was assessed as described in the Materials and Methods. As can be seen in Figure 5, incubation with Chl and RF had no impact on *A. baumannii* and *S. maltophilia*, viability indicating that the used PSs are nontoxic without irradiation.

**Figure 6:**
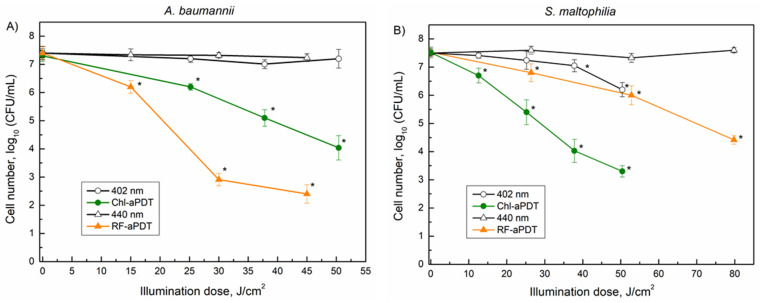
Inactivation of *A. baumannii* (**A**) and *S. maltophilia* (**B**) planktonic cells by aPDT as function of illumination doses: 402 nm and Chl-aPDT (0.015 mM Chl)—irradiance 42 mW/cm^2^ (**A**,**B**); 440 nm and RF-aPDT (0.011 mM RF)—irradiance 25 mW/cm^2^ (**A**); 440 nm and RF-aPDT (0.011 mM RF—irradiance 44 mW/cm^2^ (**B**). Every point is the average of 3–6 experiments, error bars indicate standard deviation. * indicates statistical significance compared to control culture, *p* < 0.05.

Next, the effects of 402 nm near-UV and 440 nm blue LED light on the viability of planktonic cells were analyzed. The light alone did not significantly impair *A. baumannii* viability when the illumination dose was 50.4 J/cm^2^ (402 nm) and 45 J/cm^2^ (440 nm) (Figure 6A). However, a decrease in *S. maltophilia* viability (1.3 log_10_) was apparent after the same 402 nm light exposure, indicating the photosensitivity of *S. maltophilia* SM3 isolate to near-UV light (Figure 6B).

Next, the aPDT-based inactivation of the planktonic bacterial cells was investigated. The applied illumination doses ranged from 12.6 to 79.8 J/cm^2^ using 440 nm and 402 nm LED light for the RF and Chl, respectively (Figure 6). The aPDT results showed that *A. baumannii* was more sensitive to RF-aPDT, which resulted in 5 log_10_ inactivation with the 45 J/cm^2^ illumination dose compared to Chl-aPDT, where only 3.4 log_10_ inactivation was observed after similar 50.4 J/cm^2^ of illumination (Figure 6A). Conversely, *S. maltophilia* was more sensitive to Chl-aPDT than to RF-aPDT, resulting in a decrease in CFU by 4.2 log_10_ after illumination with 50.4 J/cm^2^, whereas RF-aPDT yielded only a 1.5 log_10_ decrease in CFU under a similar 52.8 J/cm^2^ exposure (Figure 6B). Moreover, the application of a higher illumination dose of 79.8 J/cm^2^ in RF-aPDT still resulted in a lower *S. maltophilia* inactivation 3.1 log_10_ compared to the effect observed for Chl-aPDT with 50.4 J/cm^2^ (4.2 log_10_) (Figure 6B).

### 3.3. Inactivation of A. baumannii and S. maltophilia Biofilm Cells with aPDT

**Figure 7:**
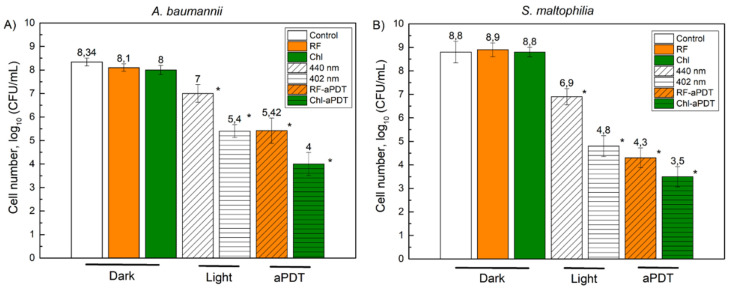
Photodynamic inactivation of *A. baumannii* (**A**) and *S. maltophilia* (**B**) biofilms using 0.11 mM riboflavin (RF) and 0.15 mM chlorophyllin (Chl) after 60 min of incubation followed by 60 min of illumination (440 nm and RF-aPDT with illumination dose 158.4 J/cm^2^; 402 nm Chl-aPDT, with illumination dose 151.2 J/cm^2^). The treatment conditions are indicated as dark, light and aPDT. CFU values present the average of 3 experiments, error bars indicate standard deviation. * shows statistical significance compared to the control culture, *p* < 0.05. The numbers above the columns are the average of log_10_ (CFU/mL).

We next investigated the efficiency of the Chl-aPDT and RF-aPDT treatments on *A. baumannii* and *S. maltophilia* biofilms (Figure 7). Bacterial biofilms are known to be more resistant to aPDT treatment [37], therefore, for inactivation of *A. baumannii*, and *S. maltophilia* biofilms we used 10 times higher PSs concentrations compared to those used for treatment of planktonic cells. In addition, to maximize adsorption of PSs to the biofilms, the microplates with formed biofilms were incubated with PS in the dark at room temperature for 60 min. Incubation with PS only did not affect the viability of biofilm bacteria, similar to the effect observed for planktonic cells of *A. baumannii* and *S. maltophilia* (Figure 7). However, light illumination using 440 nm (158.4 J/cm^2^) and 402 nm (151.2 J/cm^2^) significantly reduced cell viability. The *A. baumannii* CFU number decreased by 1.34 log_10_ and 2.94 log_10_ after the 440 nm and 402 nm light treatments, respectively. The irradiation effect was even more pronounced for the *S. maltophilia* biofilms, resulting in a reduction of CFU by 1.9 log_10_ after 440 nm irradiation and by 4 log_10_ after 402 nm light irradiation. 

RF-aPDT and Chl-aPDT resulted in a reduction of *A. baumannii* CFU numbers by 2.92 log_10_ and 4.34 log_10_, respectively (Figure 7A). Interestingly, while planktonic *A. baumannii* cells were more sensitive to RF-aPDT inactivation, biofilm bacteria were more efficiently inactivated by the Chl-aPDT. Treatment of biofilm *S. maltophilia* with RF-aPDT and Chl-aPDT resulted in a reduction of viable cell numbers by 4.5 log_10_ (158.4 J/cm^2^) and 5.3 log_10_ (151.2 J/cm^2^), respectively (Figure 7B).

## 4. Discussion

The emergence of antibiotic resistance amongst nosocomial bacterial infection agents is one of the most pressing worldwide health issues. These infection agents have developed the ability to survive in the hospital environment and gained virulent features required for efficient infection of the host [18,38]. The opportunistic pathogens *A. baumannii* and *S. maltophilia* are critically important multidrug resistant bacteria which cause severe infections to immunocompromised patients. *S. maltophilia* and *A. baumannii* are most frequently obtained from patients with pneumonia and bloodstream infections [39]. *S. maltophilia* infections are also associated with cystic fibrosis patients [40]. The ability to form biofilms is one of the most important virulence factors for both pathogens, and the majority of clinical isolates are biofilm producers [41]. It was shown that *S. maltophilia* biofilms were up to 128 times more resistant to trimethoprim/sulfamethoxazole and levofloxacin treatment compared with planktonic cells [42]. Biofilm-mediated antibiotic resistance in clinical *A. baumannii* strains was also described [43]. Therefore, as *S. maltophilia* and *A. baumannii* possess multiple resistance mechanisms and a strong capacity to form biofilms, the available options to treat infections caused by these infection agents are limited.

aPDT based on natural photoactive compounds and illumination with 402–440 nm light has the potential to be applied as an antimicrobial technique against antibiotic-resistant planktonic bacteria and biofilms [22]. The main advantages of such an approach are that there is no risk to the patients and the absence of bacterial resistance formation [44]. The interaction of PS and light in the presence of oxygen results in many cytotoxic photooxidative reactions, yielding various ROS [21,23], consequently inducing selective destruction and death of the target bacteria. The antibacterial efficacy of aPDT is influenced by many factors, especially by the physical and chemical properties of the used PSs. However, not all compounds with photosensitizing properties are suitable for aPDT [45,46]. The RF and Chl used in this study were selected due to a number of positive and suitable characteristics [22]. RF (or vitamin B2) and Chl are also known as the non-toxic food colorants E-101 and E-140ii, respectively. In addition, they have a status of “Generally Recognized as Safe” (GRAS) and are regarded as not harmful for human consumption [47]. 

RF is a neutral water-soluble compound that is heat-stable, but quickly degrades under light exposure, especially UV irradiation. RF has three absorption peaks in the UV region (at 223, 267 and 373 nm) and one peak at visible region (444 nm) [48,49]. Another relatively new PS used in this study is non-copperrized chlorophyllin sodium salt (Chl) with Mg^2+^ in the center of the ring. Chl is known as a water-soluble negatively charged (anionic) chlorophyll derivative with a main absorption maximum at 405 ± 2 nm, that exhibits antimicrobial activity that generates ROS after exposure to visible light [30,50,51]. The absorption spectra showed that RF and Chl in PBS solutions (7.4 pH) have main absorption bands at about 402 nm and 444 nm, respectively. Therefore, an LED-based combined light sourc, emitting at 440 nm and 402 nm was used in experiments for the optimal excitation of RF and Chl.

The photostability experiments helped us to assess the RF and Chl photomodifications under the conditions subsequently used to inactivate *A. baumannii* and *S. maltophilia*. It is known that during irradiation, RF in aqueous solutions is prone to degradation, resulting in various photoproducts such as formylmethylflavin, lumichrome, lumiflavin, carboxymethylflavin, 2,3-butanedione, a β-keto acid and a di-keto compound [48,49,52]. The types of photoproducts depend on the solvent, pH, buffer type, PS concentration, oxygen content, applied light intensity and spectral region [48]. We showed that irradiation of RF in PBS buffer (pH 7.4), resulted in the disappearance of absorbance peak at 444 nm and the transformation of 373 nm peak to 353 nm (Figure 4). Other studies reported same RF spectral changes under similar conditions (neutral pH and visible light irradiation) and identified lumichrome as the main RF photodegradation product [34,35,52]. Lumichrome is known to be an efficient PS [53]. Therefore, its photoactivation during RF-aPDT under chosen conditions and its subsequent contribution to bactericidal efficiency cannot be ruled out. 

In this study, we demonstrate for the first time that RF and Chl-based aPDT inactivate the opportunistic pathogens *A. baumannii* and *S. maltophilia* and their biofilms. We found that *A. baumannii* planctonic cells were highly susceptible to RF-aPDT treatment using a 0.01 mM RF concentration, yielding a reduction of viable bacteria by 5 log_10_ after exposition with 45 J/cm^2^. Maish et al. [28] showed that the positively charged RF derivatives FLASH-01a and FLASH-07a (0.01 mM), irradiated with 9 J/cm^2^ and 3 J/cm^2^ expositions (50 mW/cm^2^), reduced *A. baumannii* CFU by 5.7 log_10_ and 5.8 log_10_ using source emitting non-coherent 380–600 nm light. Another natural PS, Aloe emodin, after irradiation of 0.1 mM PS solution with 435 nm ± 10 nm (80 mW/cm^2^) at a 96 J/cm^2^ illumination dose, reduced the CFU of clinical *A. baumannii* from 4.5 to 6.89 log_10_, depending on the bacterial isolate [25]. The *A. baumannii* inactivation efficiency shown for RF-aPDT in our study, is similar to that reported for Aloe emodin, although the former was achieved applying a ten-times higher dye concentration and a twice-higher illumination dose. Chang et al. [26] reported 97.5% inhibition of imipenem-resistant *A. baumannii* planktonic cells with curcumin-based aPDT. The effect was achieved by applying a 0.2 mM PS concentration and blue light irradiation using a 5.4 J/cm^2^ illumination dose (3 mW/cm^2^). aPDT using 1.25 mM 5-ALA, a precursor of PS protoporphyrin IX, reduced the number of viable *A. baumannii* planktonic cells by 2.95 log_10_ only after 4 h preincubation with 5-ALA, followed by 402 nm blue light irradiation with 23.4 J/cm^2^ illumination dose [27]. 

The literature review showed that there are no studies analyzing inactivation of *A. baumannii* biofilms with natural PS-based aPDT. Z. Fekrirad et al. [54] analyzed the inactivation of biofilms formed by two clinical *A. baumannii* isolates by applying aPDT mediated by anionic PS erythrosine B, although only 0.1 mM erythrosine B complex with chitosan (12.5 mg/mL) and acetic acid (0.01%) with an 80 J/cm^2^ illumination dose resulted in >3 log_10_ reduction of biofilm bacteria. In our study, anionic Chl alone caused a >4 log_10_ reduction of *A. baumannii* biofilm bacteria, albeit by applying a two-times higher illumination dose with a 402 nm LED light (Figure 7A).

To the best of our knowledge, there is no published data about aPDT-caused inactivation of *S. maltophilia* pathogen. While planktonic *S. maltophilia* cells were more sensitive to Chl-aPTD compared to RF-aPDT (Figure 6B), the inactivation of biofilms using RF-aPDT and Chl-aPDT produced similar results of 4.5 log_10_ and 5.3 log_10_, respectively (Figure 7B). On the contrary, while *A. baumannii* planktonic cells were more sensitive to RF-aPDT (Figure 6A), the biofilm bacteria were more susceptible to Chl-aPDT, similarly to the inactivation of *S. maltophilia* biofilms. Interestingly, 402 nm near-UV light alone significantly reduced the viability of biofilm cells, for instance the *S. maltophilia* inactivation efficiency using 402 nm light was close to that of RF-aPDT (440 nm light) under similar illumination dose. According to our observations, more than a two-times higher illumination dose and ten-times higher PS concentrations are required to achieve the inactivation effect on *A. baumannii* and *S. maltophilia* biofilm cells, comparable to that observed for planktonic cells. Compared to RF-aPDT, Chl-aPDT has a stronger inactivation effect on biofilms of both *A. baumannii* and *S. maltophilia*, while for the planktonic cells, the inactivation results were different. *A. baumannii* was more sensitive to RF-aPDT while *S. maltophilia* was more sensitive to Chl-aPTD. RF-aPDT and Chl-aPDT may be an optional antimicrobial method for control of these pathogens. 

In summary, all the analyzed results suggest that RF-aPDT and Chl-aPDT can be used as a potential antimicrobial treatment for *A. baumannii* and *S. maltophilia* inactivation, both for planktonic and biofilm cells. Biofilm cells of both pathogens were more susceptible to near-UV light, indicating that Chl-aPDT with 402 nm LED light illumination is more suitable to inactivate *A. baumannii* and *S. maltophilia* biofilms.

## Figures and Tables

**Table 1:** Illumination doses used in experiments.

Time (min)	Illumination Dose (J/cm^2^)		
	440 nm Irradiance 25 mW/cm^2^	402 nm Irradiance 42 mW/cm^2^	440 nm Irradiance 44 mW/cm^2^
0.25	-	0.63	0.66
0.5	-	1.26	1.32
1	-	2.52	2.64
2	-	5.04	5.28
3	-	7.56	7.92
5	-	12.6	-
10	15	25.2	26.4
15	-	37.8	39.6
20	30	50.4	52.8
30	45	75.6	79.8
60	-	151.2	158.4
